# Computational study on new natural compound inhibitors of indoleamine 2,3-dioxygenase 1

**DOI:** 10.18632/aging.103113

**Published:** 2020-06-22

**Authors:** Shanshan Jiang, Hui Li, Lianhua Piao, Zheng Jin, Jingyi Liu, Sitong Chen, Luwei Lucy Liu, Yujie Shao, Sheng Zhong, Bo Wu, Weihang Li, Jiaxin Ren, Yu Zhang, Hao Wang, Rihua Jin

**Affiliations:** 1 Department of Neurosurgery, The First Hospital of Jilin University, Changchun, China; 2 Institute of Zoology, Chinese Academy of Sciences, Chaoyang, China; 3 College of Basic Medical Sciences, Jilin University, Changchun, China; 4 Clinical College, Jilin University, Changchun, China; 5 Department of Biomedical Informatics, Harvard Medical School, Boston, MA 02115, USA; 6 Department of Orthopaedic Surgery, Xijing Hospital, The Fourth Military Medical University, Xi’an, China; 7 Department of Orthopaedic Surgery, The First Hospital of Jilin University, Changchun, China; 8 Department of Neurology, The First Hospital of Jilin University, Changchun, China

**Keywords:** IDO (Indoleamine 2,3-Dioxygenase), inhibitor, virtual screening, tryptophan

## Abstract

Indoleamine 2,3-Dioxygenase (IDO), is a speed limiting enzyme that catalyzes the decomposition and metabolism of Tryptophan along Tryptophan-IDO-Kynurenine pathway [1]. Tryptophan is a necessary amino acid for activating cell growth and metabolism. Additionally, the insufficiency of Tryptophan can lead to immune system dysfunction. Raising the level of Indoleamine 2,3-Dioxygenase protein can promote stagnation and apoptosis of effector T cells [2].

In contrast, the decline in the number of effect T cells naturally protects cancer cells from attack. Therefore, Indoleamine 2,3-Dioxygenase is a potential target for tumour immunotherapy, such as melanoma, ovarian cancer, lung cancer, leukaemia, and so on, especially in solid tumours [3]. In the study, we have done sets of virtual screening aided by computer techniques in order to find potentially effective inhibitors of Indoleamine 2,3-Dioxygenase. Firstly, screening based on structure was carried out by Libdock. Then, ADME (adsorption, distribution, metabolism, excretion) and toxicity prediction were also analyzed. Molecular docking and 3D-QSAR pharmacophore generation were used to study the mechanism of these compounds and Indoleamine 2,3-Dioxygenase’s binding. A molecular dynamic analysis was carried out to assess if these potential compound’s binding is stable enough. According to the results of the analysis above, two potential compounds (ZINC000012495022 and ZINC000003791817) from the ZINC database were discovered to interact with Indoleamine 2,3-Dioxygenase with appropriate energy and proved to be none toxic. The study offered valuable information of Indoleamine 2,3-Dioxygenase inhibitor-based drug discovery in cancer therapy by increasing the activity of T cells and releasing immunity suppression [4, 5].

## INTRODUCTION

Indoleamine 2,3-Dioxygenase (IDO)is a speed limiting enzyme that activates the decomposition and metabolism of Tryptophan along Tryptophan-IDO-Kynurenine pathway [6]. Tryptophan is a necessary amino acid for cell growth and metabolism, and the deficiency of Tryptophan can lead to immune system dysfunction.

The key enzymes of Tryptophan-IDO-Kynurenine pathway include pyridoxine-2, 3-oxygenase 1 (indoleamine 2, 3-dioxygenase 1, IDO1), pyridoxine-2, 3-oxygenase 2 (indoleamine 2, 3-dioxygenase 2, IDO2), Tryptophan-2,3- Double oxygenase (Tryptophan-2, 3-dioxygenase, TDO) [7]. The activity of these enzymes can cause Tryptophan to over-produce Kynurenine, resulting in Tryptophan deficiency while producing a large amount of Kynurenine, thereby suppressing the immune system. Since Kynurenine is an endogenous tumour-promoting ligand, it binds to aromatic receptors and activates aromatic receptors to perform biological effects, which together contribute to the occurrence and development of tumours.

In the Tryptophan-IDO-Kynurenine pathway, IDO1/IDO2/TDO catalytic Tryptophan c-2/C-3 in the bond break, forming N-mention in the Kynurenine, which is not only Tryptophan-IDO-Kynurenine pathway’s first step but also the entire reaction pathway speed limit step. The downstream reaction produces metabolites such as Kynurenine, Kynurenic acid and Quinolinic acid. When the expression level of IDO1/IDO2/TDO in tissue cells increased, a large amount of Tryptophan was metabolized through the Tryptophan-IDO-Kynurenine pathway. In the meantime, Tryptophan content decreased, directly inhibiting the activation and proliferation of effect T cells. The metabolites produced by the Tryptophan-IDO-Kynurenine channel will suppress immune, reproductive and central nervous system, in addition, to directly inhibiting the survival of T-cells. Moreover, the metabolites will also promote the differentiation of regulatory T-cells and inhibit the effect of T-cells. As many studies show, Tryptophan-IDO-Kynurenine pathway plays a vital role in causing immune escape in cells [8, 9].

As for these three IDO enzymes above, their average body expression, location in the body chromosome number and tumour expression, have some differences [10]. First of all, IDO1 is coded by the ido1 gene on chromosome 8. It’s mainly expressed in the small intestine, stomach, brain and liver while expressed at lower level in other tissues of the body. Also, studies have shown that IDO1 is highly expressed in tumours such as cervical, stomach, colon and pancreatic cancer. Additionally, IDO2 is represented by the ido2 gene on chromosome 8, which is not far from the ido1 gene. It has a substantial similarity to IDO1 at amino acid levels. However, IDO2 is not widely expressed than IDO1. And IDO2 is mainly expressed in the kidney, liver, testicles, appendicitis and other tissues. Also, according to studies, IDO2 is over-expressed in colon cancer, stomach cancer, kidney and pancreatic cancer. Moreover, TDO is encoded by the TDO2 gene located on chromosome 4. And it is mainly expressed in the liver in the human body [11]. TDO in glioma is high. And in recent years, many studies have reported that TDO is over-expressed in breast, rectal, and lung cancer. There have been many studies and inhibitor-drugs targeting IDO1, while IDO2 and TDO research is relatively rare.

The IDO1 protein consists of a large and small domain with the active site located in a larger area. And the contact surfaces of the two fields form two hydrophobic pockets, S1 and S2. The main S1 bag consists of Tyr126, Val130, Phe163, Phe164, Leu234, while the S2 bag owner consists of Phe226, Phe227, Arg231, Ile354. The amino acid residue of these two pockets and hemoglobin are essential for the binding and selectivity of IDO1 inhibitors.

Kynurenine is toxic and can lead to apoptosis of lymphocytes [12]. More than 95% Tryptophan is oxidized and metabolized along Tryptophan-IDO-Kynurenine pathway to produce Kynurenine and its derivatives like quinolinic acid and Kynurenic acid, etc. Among them, quinolinic acid is the starting material of the NAD+(nicotinamide adenine dinucleotide) synthesis pathway. On the one hand, NAD+ is an indispensable substance in the electron transmission process and plays a vital role in promoting energy metabolism and signal transduction. In addition, NAD+ plus its prototype NADH is essential to maintaining the reductive environment within the cell and thus protecting the cells from oxidative stress. Therefore, sufficient NAD+ in cells is a critical factor in protecting cells. Additionally, tumour cells have an uncontrolled proliferation rate, rapid metabolic rate, and significantly increased levels of oxidative stress, compared to healthy cells, making tumour cells more sensitive to changes in NAD+ levels. Key enzymes in the biosynthesis pathway in NAD+ have become important targets for anti-cancer drugs. On the other hand, excessive expression of IDO was found in many cancers as reported, such as prostate cancer, pancreatic cancer, breast cancer, stomach cancer. Increasing IDO will cause depletion of local Tryptophan and increase of Kynurenine and other metabolites. Additionally, increasing IDO can also inhibit the proliferation of T cells and activate regulated T cells, and thus cause immune tolerance. So that the body can not identify and remove tumour cells. Therefore, developing potential inhibitors of IDO could offer more effective therapeutic methods for metabolic diseases and cancers [13, 14].

Numerous studies have shown that abnormal cell metabolism is one of the most critical signs of cancer [15]. Recently, IDO has been reported to have lots of small-molecule drugs by inhibiting IDO’s activities through Tryptophan-IDO-Kynurenine pathway. For example, Indoximod inhibits IDO’s activities indirectly by relieving T-cell functional inhibition [16]; Epacadostat has high selectivity to IDO; Navoximod is TDO/IDO1’s dual inhibitor; BMS-986205 is an irreversible inhibitor with non-hemoglobin binding activity; PF-06840003 is a non-competitive inhibitor with non-heme binding activity [17]. Among these drugs, Incyte’s Epacadostat is an oral, practical, and selective small-molecule IDO inhibitor. The development of this drug is currently in the clinical stage of Phase 3 and has achieved significant results in the clinical stage of Phase 2. Thus, as for the reference drug, we chose Epacadostat [18–21]. Additionally, the over-expression of IDO can lead to the over-production of “neurotoxin” quinolinic acid, which can eventually cause damage to neurons and neurological diseases such as Alzheimer’s disease. Thus, much of the focus on IDO was on its relationship to Alzheimer’s disease and neurological disorders previously. It can be seen that if the excessive expression of IDO is curbed, it will be beneficial to the treatment of cancer and Alzheimer’s diseases. Therefore, IDO inhibitors are a potential new target drug molecule [23]. Moreover, there have not been IDO inhibitor drugs in the world, so it matters a lot to do much more study and research on IDO inhibitor.

According to reports in recent years, natural products have played a vital role in both molecular biological research and potential drug study. To find new potential IDO inhibitors, a virtual screening were carried out against the Natural Products database (NP) in the ZINC database. Then, ADME (absorption, distribution, metabolism, excretion) and toxicity properties were analyzed and assessed. Binding modes and interactions between potential compounds and IDO were analyzed by docking. And molecular dynamics simulation was utilized to assess if their binding interactions are stable. This study greatly promoted the development of IDO inhibitors for providing potential inhibitor drugs and their drug-like properties.

## RESULTS

### Virtual screening of potential stimulators of Indoleamine 2,3-Dioxygenase, IDO

Area of the ligand with iron hemoglobin is an essential regulatory site of IDO. As studies show, part of small molecules can interact with IDO well in this region. Then, this binding can inhibit IDO’s activities of catalyzing the decomposition and metabolism of Tryptophan along Tryptophan-IDO-Kynurenine pathway, and then block the T cells from being destroyed. So, we chose this site as the reference region. Firstly, we used Libdock to virtually screen favorable small molecules of IDO by DS4.5 (Discovery Studio 4.5, Accelrys, Inc., San Diego, CA, USA). Twenty-five thousand nine hundred thirty-two natural and purchasable molecules were got from this database. ZINC database is commercially free. Moreover, Irwin and Shoichet Laboratories in the Department of Pharmaceutical Chemistry at UCSF (the University of California, San Francisco) provides the compounds of ZINC database. The target used in this study was the 3D structure of IDO. The well known IDO inhibitor Epacadostat can inhibit IDO activity in vitro as well as in vivo. So we chose Epacadostat as the reference compound. Epacadostat suppressed IDO by interacting with and binding to iron hemoglobin of IDO by inserting into this ligand pocket. The inhibition of this distinct resulted in inhibition of IDO activities by inhibiting the decomposition and metabolism of Tryptophan, which is a necessary amino acid for cell growth and metabolism, and it is an essential amino acid related to immune system dysfunction [24, 25]. After the screening, 247 compounds were found to have higher Libdock scores than Epacadostat (Libdock score: 119.882). The top 20 ranked compounds are listed in Table 1.

**Table 1:** Top 20 ranked compounds with higher Libdock scores than Epacadostat.

**Number**	**Compounds**	**Libdock score**	**Number**	**Compounds**	**Libdock score**
1	ZINC000056897657	185.52	11	ZINC000013540662	163.201
2	ZINC000035024527	172.106	12	ZINC000002528510	162.775
3	ZINC000118912913	169.516	13	ZINC000049878068	162.665
4	ZINC000038559596	167.201	14	ZINC000003791817	161.856
5	ZINC000013540213	167.102	15	ZINC000100822245	161.737
6	ZINC000034944431	166.571	16	ZINC000017611788	161.466
7	ZINC000012495022	165.456	17	ZINC000012496925	159.247
8	ZINC000002526389	165.297	18	ZINC000008219593	158.961
9	ZINC000100013581	165.169	19	ZINC000032821825	156.629
10	ZINC000085886023	164.103	20	ZINC000012496524	153.431

### Assessment of drug-like properties for Absorption, Distribution, Metabolism, Excretion (ADME) and toxicity

**Figure 1:**
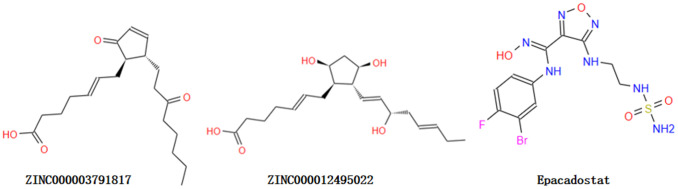
**The structures of ABZI and novel compounds selected from virtual screening by chemdraw.**

Through ADME module of Discovery Studio 4.5, we assessed drug-like properties of Epacadostat and favorable compounds, including BBB (brain/blood barrier), human intestinal absorption, aqueous solubility, CYP2D6 (cytochrome P450 2D6) binding, PPB (hepatotoxicity and plasma protein binding properties) (Table 2). The aqueous solubility assessing was carried out with suitable temperature of water at 25°C. As the results shown, all the compounds can dissolve well in water other than ZINC000118912913. 10 compounds had favorable solubility and Epacadostat had moderate solubility. For human intestinal absorption, 7 compounds and Epacadostat were absorbed with a secondary level, and 10 compounds were absorbed with a right level. And we find 12 compounds and Epacadostat interacted and combined with plasma protein tightly, and the rest was not. All compounds don’t inhibit cytochrome P450 2D6 (CYP2D6) other than ZINC000034944431, ZINC000002526389, ZINC000085886023, ZINC000002528510 and ZINC000100822245. Cytochrome P450 2D6 (CYP2D6) played a vital part in metabolism of drug. Epacadostat were predicted to be non-inhibitors of cytochrome P450 2D6 (CYP2D6) as well. For hepatotoxicity, 16 compounds were predicted as non-toxic, while the rest and Epacadostat were toxic. Then, though TOPKAT module of Discovery Studio 4.5, we also assessed Safety properties of the selected compounds and Epacadostat, including AMES, DTP and Rodent carcinogenicity. AMES is Ames mutagenicity. DTP is developmental toxicity potential. And Rodent carcinogenicity is based on the U.S. National Toxicology Program (NTP) dataset (Table 3). As the results shown, 9 compounds were not toxic in development. All results above indicated that compound 1 (ZINC000012495022) and compound 2 (ZINC000003791817) were favorable potential compounds as inhibitors of IDO. These two compounds above didn’t suppress CYP2D6’s activities and were not hepatotoxic. Moreover, they were almost not mutagenic, rodent carcinogenic and developmental toxic with the comparison of others. So these two selected compounds can be perspective target of inhibitor drug development for IDO. As Figure 1 shown, these two compounds and Epacadostat were similarly made up of several multiple reactive oxygens, dual-band in their chemical structures. And compound 1 (ZINC000012495022) and compound 2(ZINC000003791817)’s constructions were similar to Epacadostat. Moreover, the two candidate compounds and Epacadostat both bind with IDO at a same position, which is close to IDO’s ligand iron hemoglobin. In summary, these two compounds were safe. So we chose them as potential candidate compounds for following study (Figure 1).

**Table 2:** ADME (Adsorption, Distribution, Metabolism, Excretion) properties of compounds.

**Number**	**Compounds**	**Solubility level**	**BBB level**	**CYP2D6^c^**	**Hepatotoxicity^d^**	**Absorption level^e^**	**PPB level^f^**
1	ZINC000056897657	1	4	0	0	3	0
2	ZINC000035024527	1	4	0	0	3	0
3	ZINC000118912913	0	4	0	0	3	1
4	ZINC000038559596	3	4	0	0	2	0
5	ZINC000013540213	3	3	0	0	0	1
6	ZINC000034944431	2	4	1	0	2	0
7	ZINC000012495022	3	1	0	0	0	1
8	ZINC000002526389	2	4	1	1	0	1
9	ZINC000100013581	3	2	0	0	0	1
10	ZINC000085886023	1	4	1	1	3	1
11	ZINC000013540662	4	4	0	0	1	0
12	ZINC000002528510	2	4	1	1	0	1
13	ZINC000049878068	1	4	0	0	3	0
14	ZINC000003791817	4	3	0	0	0	1
15	ZINC000100822245	2	4	1	0	3	0
16	ZINC000017611788	2	4	0	1	3	0
17	ZINC000012496925	4	3	0	0	0	1
18	ZINC000008219593	3	1	0	0	0	1
19	ZINC000032821825	3	1	0	0	0	1
20	ZINC000012496524	3	3	0	0	0	1
21	Epacadostat	2	4	0	1	3	1

^a^Aqueous-solubility level: 0 (extremely low); 1 (very low, but possible); 2 (low); 3 (good).

^b^Blood Brain Barrier level: 0 (Very high penetrant); 1 (High); 2 (Medium); 3 (Low); 4 (Undefined).

^c^Cytochrome P450 2D6 level: 0 (Non-inhibitor); 1 (Inhibitor).

^d^Hepatotoxicity: 0 (Nontoxic); 1 (Toxic).

^e^Human-intestinal absorption level: 0 (good); 1 (moderate); 2 (poor); 3 (very poor).

^f^Plasma Protein Binding: 0 (Absorbent weak); 1 (Absorbent strong).

**Table 3:** Toxicities of compounds.

**Number**	**Compounds**	**Mouse NTP^a^**		**Rat NTP^a^**		**AMES^b^**	**DTP^c^**
		**Female**	**Male**	**Female**	**Male**		
1	ZINC000056897657	0	1	1	0.002	1	1
2	ZINC000035024527	1	0.727	1	0	0	1
3	ZINC000118912913	1	1	0.996	1	0	1
4	ZINC000038559596	0	0.001	0.802	0	0.434	1
5	ZINC000013540213	0	0	0	0	0.434	0.002
6	ZINC000034944431	0	0	1	0.02	0	1
7	ZINC000012495022	0	0	0	0	0.001	0
8	ZINC000002526389	0.999	0.036	0	0.999	1	0.769
9	ZINC000100013581	0.095	0	0	0.001	0.051	0.989
10	ZINC000085886023	0	0.003	0	1	0	0.989
11	ZINC000013540662	0	0	0	0	0.051	0
12	ZINC000002528510	0.999	0.036	0	0.999	0	0.769
13	ZINC000049878068	0.837	0.938	0	1	0.999	0.906
14	ZINC000003791817	0	0	0	0	0.019	0
15	ZINC000100822245	0	1	1	0.051	0.434	1
16	ZINC000017611788	0.075	0.929	1	0	0	1
17	ZINC000012496925	0	0	0.12	0	0.135	0.015
18	ZINC000008219593	1	0	0	0.024	0.976	0
19	ZINC000032821825	0	0.001	0	0	0	0
20	ZINC000012496524	0	0	0.002	0	0.014	0.005
21	Epacadostat	0	0	0.035	0.001	0	0.047

^a^<0.3 (Non-Carcinogen); >0.7 (Carcinogen).

^b^<0.3 (Non-Mutagen); >0.7 (Mutagen).

^c^<0.3 (Non-Toxic); >0.7 (Toxic).

### Ligand binding analysis and ligand pharmacophore

**Figure 2:**
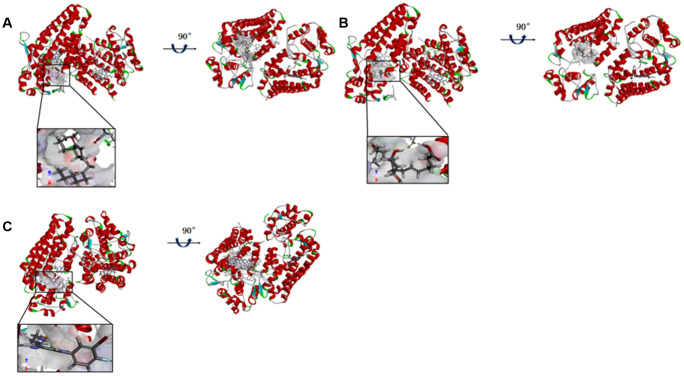
(**A**) ZINC000012495022-IDO complex. Schematic drawing of interactions between ligands and IDO, the surface of binding area were added, blue represented positive charge, red represented negative charge, and ligands were shown in sticks, the structure around the ligand-receptor junction were shown in thinner sticks. (**B**) ZINC000003791817-IDO complex. Schematic drawing of interactions between ligands and IDO, the surface of binding area were added, blue represented positive charge, red represented negative charge, and ligands were shown in sticks, the structure around the ligand-receptor junction were shown in thinner sticks. (**C**) Epacadostat-IDO complex. Schematic drawing of interactions between ligands and IDO, the surface of binding area were added, blue represented positive charge, red represented negative charge, and ligands were shown in sticks, the structure around the ligand-receptor junction were shown in thinner sticks.

**Figure 3:**
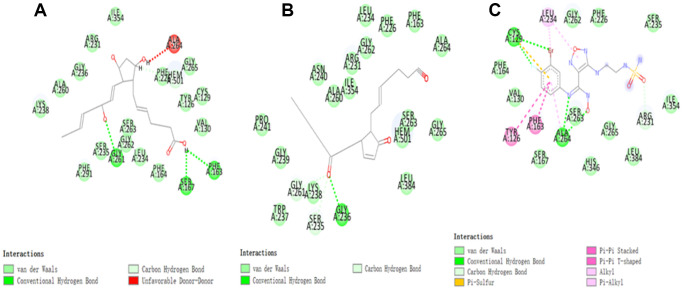
The inter-molecular interaction of the predicted binding modes of (**A**) ZINC000003791817 to IDO; (**B**) ZINC000012495022 to IDO, (**C**) Epacadostat to IDO.

**Figure 4:**
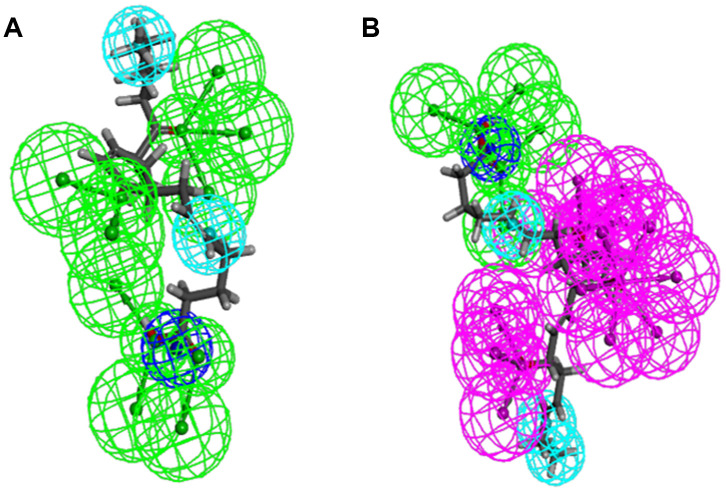
**Pharmacophore predictions using 3D-QSAR.** (**A**) ZINC000012495022: Green represents hydrogen acceptor, and blue represents hydrophobic center and dark blue represents Ionizable negative. (**B**) ZINC000003791817: Green represents hydrogen acceptor, blue represents hydrophobic center, purple represents hydrogen donor, and dark blue represents Ionizable negative.

As the results shown, the RMSD (Root Mean Square Deviation) was 0.6Å, which was between the docked pose and the crystal structure of the complex. So the CDOCKER module in this study was very reliable. CDOCKER module was applied under CHARMm36 force field. We have docked compounds 1,2 into IDO by CDOCKER module. As the results (Tables 4) shown, Epacadostat (- 46.2081kcal/mol) has higher CDOCKER potential energy than the two selected compounds. So, compounds 1, 2 might bind with IDO with higher affinity than the reference ligand Epacadostat. Structural assessment between ligands and IDO, such as hydrogen bonds and Pi-S interactions were also carried out. As shown in Figures 2, 3 and Tables 5 and 6, compound 1 formed no Pi-S interaction. It formed only one pair of hydrogen bond with IDO (A:GLY236: HN - ZINC000012495022: O7) in the complex. Compound 2 had three hydrogen bonds with IDO (A:GLY261: HN- ZINC000003791817: O7, A:SER167: OG- ZINC000003791817: H57, A: PHE163:O- ZINC000003791817: H57). There was also no Pi-S interaction formed within the complex. Moreover, Epacadostat created four hydrogen bonds with IDO, (A:CYS129: SG- Epacadostat: F7, A:CYS129: SG- Epacadostat: Br8, A:ALA264: N9- Epacadostat: O12, A:ALA264: N9- Epacadostat: N9 independently). Also, Epacadostat formed two pairs of Pi-Pi and one pair of Pi-S interactions with IDO. ZINC000012495022 displayed hydrogen bond acceptor, hydrophobic center and Ionizable negative. ZINC000003791817 revealed hydrogen bond acceptor, hydrogen bond donor, hydrophobic center, and Ionizable negative in computational prediction of pharmacophore (Figure 4).

**Table 4:** CDOCKER interaction energy of compounds with Indoleamine 2,3-Dioxygenase, IDO.

**Compounds**	**CDOCKER interaction energy (Kcal/mol)**
ZINC000012495022	- 51.3317
ZINC000003791817	- 49.7711
Epacadostat	- 46.2081

**Table 5:** Hydrogen bond interaction parameters for each compound and IDO residues.

**Receptor**	**Compound**	**Donor atom**	**Receptor atom**	**Distances (Å)**
IDO	Epacadostat	A:CYS129: SG	Epacadostat: F7	3.09
		A:CYS129:SG	Epacadostat:Br8	2.62
		A:ALA264:N9	Epacadostat:O12	3.11
		A:ALA264:N9	Epacadostat:N9	2.83
	ZINC000003791817	A:GLY261: HN	ZINC000003791817: O7	2.52
		A:SER167: OG	ZINC000003791817: H57	2.04
		A: PHE163:O	ZINC000003791817: H57	3.01
	ZINC000012495022	A:GLY236:HN	ZINC000012495022:O7	2.01

**Table 6:** Pi-S and Pi-Pi interaction parameters for each compound and IDO residues.

**Interaction parameters**	**Receptor**	**Compound**	**End1**	**End2**	**Distances (Å)**
	IDO	Epacadostat	A: PHE163	Epacadostat: O2	4.46
Pi-Pi interaction			A: TYR126	Epacadostat: O2	5.21
Pi-S interaction			A:CYS129:SG	Epacadostat:O2	5.90

### Molecular dynamics simulation

**Figure 5:**
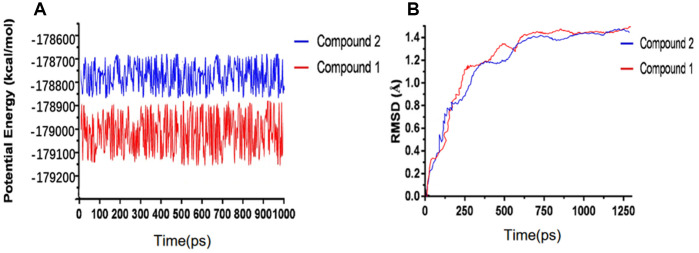
**Results of molecular dynamics simulation of three complexes.** (**A**) Potential Energy; (**B**) Average backbone RMSD.

We have performed molecular dynamics simulation to assess if the complex of ligand-IDO is stable under natural environment. The results included RMSD curves and potential energy profiles (Figure 5). The RMSD curves of these complexes got equilibrium at about 200 ps. Moreover, RMSD as well as potential energy of these complexes got stable gradually. As results shown, hydrogen bonds and pi-s interactions between compounds and IDO greatly promoted these complex’s stability. So the complex of these two selected compounds and IDO can keep stable and have promotional effects under natural environment as Epacadostat did.

### Experimental results to validate the effectiveness of the compounds

**Figure 6:**
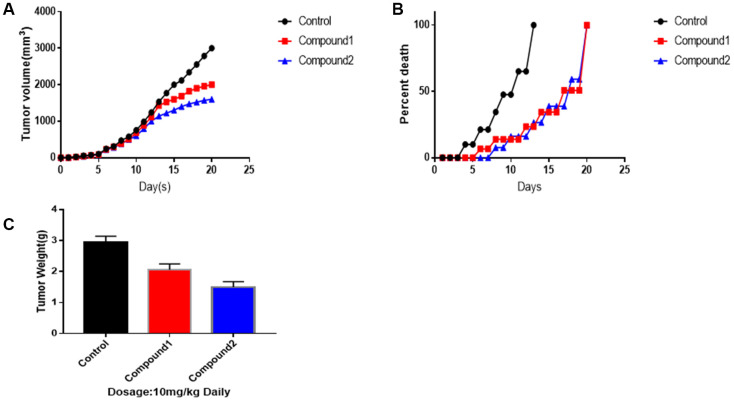
**Animal experiments to against tumor activity. Tumor-bearing mice were treated with compound 1,2 at dosage of 10 mg/kg, respectively.** (**A**) Mean tumor volumes. (**B**) Survival percentage of Mice. (**C**) Tumor weights on 20^th^ day. Data were represented as Mean ± SEM, p<0.05 and p<0.01.

To make the results of the two selected compounds more convincing (Figure 6), we performed animal experiments. As figure 6 showed to us, compound 1 and 2 had a great advantage to suppress tumour growth, both in volume and weight, especially compound 2. After 12^th^ day, compounds 1 and 2 made a significant difference in killing the tumour. As for tumour volumes, on 20^th^ day, the control group was 3014 mm^3^, the tumour volumes treated by compounds 1 and 2 were 2010, 1560 mm^3^, independently (mean ± SEM, p<0.05). Additionally, as survival per cent chart shown, compounds 1 and 2 play an essential role in promoting survival period. What’s more, on 20^th^ day, as tumour weight chart illustrated, control group’s final weight of tumour was 3.18g. Tumour weight treated by compounds 1 was 2.05g. Tumour weight treated by compounds 2 was 1.54g (mean ± SEM, p<0.01).

## DISCUSSION

Tryptophan plays a significant role in maintaining the stability of the internal and immune environment, which is not only Immune cell nutrition factor but also the Signal conduction factor. The Tryptophan-IDO-Kynurenine pathway is Tryptophan’s major metabolic pathways. Over-expression of IDO elicits degradation of Tryptophan to Kynurenine, which inhibits the effector T-cells from attacking the function of cancer cells and promotes the immune suppression function of T-cells. Moreover, Kynurenine is toxic and can cause the apoptosis of lymphocytes. Among Tryptophan’s derivatives, quinolinic acid is the starting material of the NAD+ synthesis pathway. In addition, NAD+ plus its prototype NADH is vital for maintaining the reductive environment within the cell and thus protecting the cells from oxidative stress. And tumour cells have an uncontrolled proliferation rate, rapid metabolic rate, and significantly increased levels of oxidative stress, compared to healthy cells. Thus, tumour cells more sensitive to changes in NAD+ levels. A study has reported that there are significant over-expression of IDO in infection or cancer. Consequently, immune response was suppressed to protect the body from disease or bacteria. Therefore, it’s of great importance to suppress Tryptophan-IDO-Kynurenine pathway and accelerate T-cell proliferation and activation in cancer immunotherapy. Tryptophan was only thought to be a nutrient initially. Afterwards, during macrophages/T cell culture by Munn, scientists started learning that Tryptophan may not be a mere nutrient, but more likely to be a signalling molecule.

Thus, as the study reported, IDO is an essential and promising therapeutic target.

Currently, many researches revealed that IDO played a critical pathological role and molecular biological role in Tryptophan-IDO-Kynurenine pathway and suppression of IDO. So far, there have been many drugs on Tryptophan-IDO-Kynurenine channel designed and developed with a different mechanism. For example, Indoximod inhibits IDO’s activities indirectly by relieving T-cell functional inhibition; Epacadostat has high selectivity to IDO [26]; Navoximod is TDO/IDO1’s dual inhibitor [27]; BMS-986205 is an irreversible inhibitor with non-hemoglobin binding activity; PF-06840003 is a non-competitive inhibitor with non-heme binding activity. Among these drugs, Incyte’s Epacadostat is an oral, practical, and selective small-molecule IDO inhibitor. The development of this drug is currently in the clinical stage of Phase 3 and has achieved significant results in the clinical stage of Phase 2. Thus, we chose Epacadostat as the reference drug. Furthermore, more study about developing IDO inhibitor drugs are critically needed for molecular immunotherapy. In this study, we used Libdock, ADME/TOPKAT, CDOCKER/3D-QSAR pharmacophore generation, and Molecular Dynamics Simulation, to select potential compounds and assess their drug-like properties [28, 29]. Additionally, molecular conformation, pharmacological properties, binding affinity and stability were also assessed for further selection [30, 31]. We got 25932 natural and purchasable product molecules from the ZINC15 database to screen favorable compounds virtually. The higher the Libdock score was, the more optimized the compound’s energy was, and the more stable the compound’s conformation was. As the Libdock’s results shown, 1729 compounds were found to have favorable binding and interaction with IDO. What’s more, 247 compounds had better Libdock results than Epacadostat (Libdock score: 119.882, ranking:248). So they had higher affinity and stability than Epacadostat [32, 33]. According to results above, the top 20 compounds were chosen for further study.

Drug-like properties, ADME (absorption, distribution, metabolism, excretion) and toxicity properties were assessed consequently. According to results, we chose compounds 1 and 2 as the most potential compounds. Both of them have remarkable solubility in water and outstanding absorption level. At the same time, they didn’t suppress the activities of cytochrome P450 2D6 (CYP2D6). So they had no hepatotoxicity. Furthermore, these two selected compounds were less toxic in Ames mutagenicity test, rodent carcinogenicity and developmental toxicity potential than other compounds. So these two compounds had great potential in drug study and development. On the other hand, part of the compounds which were not selected in this study also had potential application in drug study and development. Even though some of them possessed toxicity or had bad absorption, distribution, metabolism, excretion, solubility in water, atoms could be added to reduce its toxicity and improve absorption, distribution, metabolism, excretion, solubility in water. All the results above indicated that compounds 1 and 2 were ideal compounds for drug development. So we performed next study for them. We also assessed the binding and the interactions between selected compounds and IDO.

The lower the CDOCKER interaction energy was, the more affinity they have between compounds and IDO. As the CDOCKER module computation demonstrated, CDOCKER interaction energy of compounds 1 and 2 was remarkably lower than Epacadostat (- 46.2081kcal/mol). So these two selected compounds might bind with IDO with higher affinity than Epacadostat. Next, the molecular structural inspection indicated that compound 1, 2 and Epacadostat were similarly made up of several multiple reactive oxygens, dual-band in their chemical structures. And we found compound 1 (ZINC000012495022) and compound 2(ZINC000003791817) had similar construction to Epacadostat, such as axisymmetric structure. Moreover, the two candidate compounds and Epacadostat both bind with IDO at a same position and distinct, which are close to IDO’s ligand iron hemoglobin. In summary, these two compounds were safe. Thus, we chose them for the next research [34].

Regarding compound designation, computation results showed 11 feature pharmacophores in ZINC000012495022, with hydrogen bond acceptor, hydrophobic center and Ionizable negative, and 38 feature pharmacophores in ZINC000003791817, with hydrogen bond acceptor, hydrogen bond donor, hydrophobic center, and Ionizable negative in computational prediction of pharmacophore, which means these 2 compounds may have a more appropriate effective pharmacophore. Different functional groups could be added into these 2 lead compounds to increase their significance in cancer-therapy drug therapy and drug improvement in future research.

Next, we assessed their stability through molecular dynamics simulation. According to RMSD curves and potential energy of the ligand-IDO complexes, the RMSD curves of these complexes got equilibrium at about 200 ps. Moreover, RMSD as well as potential energy of these complexes got stable gradually. So the complex of these two selected compounds and IDO can keep stable and have promotional effects under natural environment as Epacadostat did.

Finally, an animal experiment was performed to make the results of the two selected compounds more convincing. And these two compounds were proved to make a significant difference in suppressing and killing the tumour. After 12^th^ day, as survival per cent chart also shown, compounds 1 and 2 play an essential role in promoting survival period. Therefore, compound 1 and 2 are promising target drugs for IDO inhibitors.

Last but not least, this study tried to find more candidates for targeted inhibitor drugs to IDO. Identified compounds in this study could remarkably promote IDO related cancer-therapy drugs’ development. Although this study had been performed with thoroughly design, we still have to admit this study has some limitations. On the one hand, more experiments are needed to validate our results and more indicators to assure drug safety. On the other hand, we can add some necessary groups to modify the drugs to improve its pharmacological properties, such as reducing toxicity, improve absorption, etc. After modification, they could be more perfect as potential inhibitor [35].

## CONCLUSIONS

We had applied a series of structural biology and chemistry method to screen and identify the potential and effective inhibitor drugs of IDO. To summarize, compounds 1 and 2 were both potential inhibitors to IDO. Furthermore, these compounds were safe and had remarkably importance in targeting inhibitor drug’s development of IDO. Moreover, this study provided lots of materials and the therapeutic basis for IDO inhibitor’s research and research.

## MATERIALS AND METHODS

### Docking software and ligand library

Discovery Studio, a software, which was designed as a system to simulating small molecule and macromolecule systems, plays a significant role in selecting the appropriate therapy-target drugs according to the chemical structure and biological computation. First of all, Libdock, along with ADME which includes absorption, distribution, metabolism and excretion, was used to screen appropriate molecules virtually. Additionally, CDOCKER was designed to assess the docking between the particles. As for our study, IDO inhibitors were downloaded from the Natural Products (NP) database in the ZINC15 database. ZINC15 database is free of commercially-available compounds (provided by the Irwin and Shoichet Laboratories in the Department of Pharmaceutical Chemistry at the University of California, San Francisco (UCSF)).

### Structure-based virtual screening using Libdock

**Figure 7:**
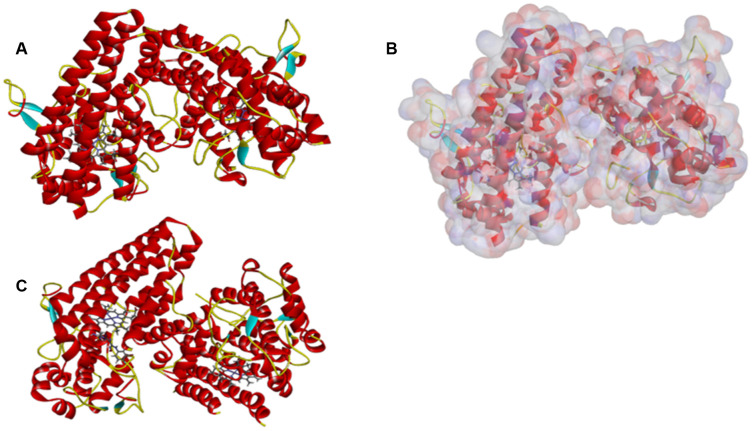
**The molecular structure of IDO.** Initial molecular structure was shown in (**A**) the surface of binding area were added in (**B**) and the complex structure of IDO with Epacadostat in (**C**) blue represented positive charge, red represented negative charge and green was used to label the cartoon.

The site where the IDO’s ligand iron hemoglobin binds with IDO was located and then was selected as the sphere binding site to screen virtually the potential appropriate molecules. Libdock is a rigid-based docking program which can assess the hotspots of the complex with a grid located at the interaction and binding region, polar and a polar probe. The hotspot is then applied to coordinate the ligand to make a favorable binding. Additionally, the Smart Minimiser algorithm and the CHARMm force field (Cambridge, MA, USA) were carried out for ligand’s minimization. Then, based on the ligand score, all ligand poses are ranked accordingly. We downloaded the complex, IDO’s 2.45Å crystal structure with Epacadostat (PDB ID: 6E40), from the PDB (Protein Database) and then performed libdock study. Figure 7 showed the chemical structure of IDO. Proteins are modified by removing water of crystallization and other heteroatoms other than the IDO’s ligand iron hemoglobin, then followed by adding hydrogen, protonation, ionization, and energy minimization. The CHARMm force field, as well as the Smart Minimiser algorithm, is used for energy minimization. Furthermore, minimize the execution of 2000 steps with an RMS (root mean square) gradient tolerance of 0.1 and a final RMS gradient of 0.09778. After all the above preparations, the protein is applied to define the binding site, and the binding site of IDO’s ligand iron hemoglobin is selected as a favourable docking site. Afterwards, Libdock is used for virtual screening by docking all of the prepared ligands to defined active sites. Then, all docked poses are sorted and grouped by compounds’ name according to the Libdock score.

### ADME (Absorption, Distribution, Metabolism, Excretion) and toxicity prediction

The ADME module of DS 4.5 is used for the calculation of absorption, distribution, metabolism, and excretion of compounds chosen. Additionally, TOPKAT (Toxicity Predicted by Komputer Assisted Technology) is also applied to assess potential compounds’ drug- like properties, such as toxicity, water-solubility, BBB (blood-brain barrier) penetration, CYP2D6 (Cytochrome P450 2D6) inhibition, human intestinal tract Absorption, PPB (plasma protein binding) levels, hepatotoxicity, rodent carcinogenicity, developmental toxicity potential, Ames Mutagenicity. These pharmacological effects and characteristics will be taken into consideration when a potential therapeutic drug of IDO inhibitor is selected.

### Molecular docking and pharmacophore prediction

CDOCKER was applied for precisely docking study of molecules which is based on CHARMm36 force field. During the docking process, the receptor remains rigid while the ligands are very flexible. Also, the CHARMm energy as well as the interaction energy of each complicated pose were calculated and assessed. These two results of energy indicated ligand binding affinity. We obtained the crystal structure of IDO from the protein data bank. Firstly, the crystal water molecules which might influence the combination between the receptor and ligand were almost deleted in the rigid and semi-flexible docking process. Additionally, the water molecules were also removed. At the same time, we added hydrogen atoms to the protein. Furthermore, to make the binding mode more reliable, Epacadostat was extracted and then re-paired into the crystal structure of IDO. The CHARMm36 force field was used for receptors well as ligands. The binding site sphere of IDO was selected as the region that came within radius 16Å from the geometric centroid of the ligand IDO. In the docking process, the ligands combined with residues within the binding site spheres. The parts of identified hits were determined and then docked into the interaction region of IDO. When the CDOCKER process was finished, each ligand displayed ten docking poses, among which, the one with the highest docking scores and appropriate docking orientations was selected. Moreover, CDOCKER interaction energy of different poses was also calculated. In addition, 3D-QSAR pharmacophore generation module was used to display the pharmacophore of the compounds, which produces up to 255 confirmations per molecule to represent small molecules. However, only conformations whose energy values were within the energy threshold of 10 kcal/mol were retained.

### Molecular dynamics simulation

Among different binding conformations of each compound-IDO complex, only the best were chosen for further molecular dynamics simulation. The ligand-receptor complex was placed in an orthorhombic box. And then it was solvated using an explicit periodic boundary solvated water model. Additionally, with the aim to assess the physiological environment, solid chloride was put into the system with an ionic strength of 0.145. The system was then applied to the CHARMm forcefield. And then it’s relaxed by energy minimization (500 steps of steepest descent and 500 steps of the conjugated gradient). The final RMS gradient was 0.289. The temperature of the system was gradually increased from 50 K to 300 K for 200 ps and a balanced simulation of 250 ps. Time step was set as 2fs. The simulation was applied using the NPT (normal pressure and temperature) system with a constant temperature of 300 K. Long-range electrostatics was calculated using the particle mesh Ewald (PME) algorithm, and the linear constraint solver (LINCS) algorithm was changed accordingly to fix all bonds involving hydrogen. Using initial sophisticated setting as a reference, structural properties, RMSD (root-mean-square deviation), and potential energy, the trajectory was determined by using trajectory protocol in Discovery Studio 4.5 (San Diego, CA, USA).

### Animal experiments to verify the effectiveness of the compound

These two selected compounds and twenty mice were provided by the Animal Experiment base in a clinical college of Jilin University. Additionally, experimental protocols were offered by Jilin University Ethics Committee. A total of 105 Colon melanoma cells were injected into the left flank of each mouse to make tumour models with the parameter of 26 cells per 100 μl PBS. Randomly, mice were then divided into three groups: (a) control group injected by only tumour cells; (b) Group treated with tumour injection as well as compound 1, at dosage of 10 mg/kg, (c) Group treated with tumour injection as well as compound 2, at dosage of 10 mg/kg. Firstly, Colon melanoma cells were injected. Then, after four hours, the tumour-bearing mice were treated with compound 1,2 injection through tail. These two steps were repeated every day, 20 days total. Additionally, we measured tumour volumes and weight every day. Each group had 7 mice. And we averaged the final results to make the results more reasonable and convincing. On day 20^th^, all survival mice were counted, then measured and sacrificed. Finally, tumours were all removed and weighed.

## References

[1] Gao J, Deng F, Jia W. Inhibition of Indoleamine 2,3-Dioxygenase Enhances the Therapeutic Efficacy of Immunogenic Chemotherapeutics in Breast Cancer. J Breast Cancer. 2019; 22:196–209. 10.4048/jbc.2019.22.e23 31281723 PMC6597411

[2] Nepal P, Mori S, Kita Y, Tanabe K, Baba K, Sasaki F, Nasu Y, Ido A, Uchikado Y, Kurahara H, Arigami T, Sakoda M, Maemura K, Natsugoe S. Combined endoscopic submucosal dissection and transanal minimally invasive surgery for the management of lower rectal adenoma extending above the dentate line: A case report. Medicine (Baltimore). 2019; 98:e15289. 10.1097/MD.0000000000015289 31083160 PMC6531252

[3] Winters M, DuHadaway JB, Pham KN, Lewis-Ballester A, Badir S, Wai J, Sheikh E, Yeh SR, Prendergast GC, Muller AJ, Malachowski WP. Diaryl hydroxylamines as pan or dual inhibitors of indoleamine 2,3-dioxygenase-1, indoleamine 2,3-dioxygenase-2 and tryptophan dioxygenase. Eur J Med Chem. 2019; 162:455–64. 10.1016/j.ejmech.2018.11.010 30469041 PMC6318801

[4] Wang Q, Ding Y, Song P, Zhu H, Okon I, Ding YN, Chen HZ, Liu DP, Zou MH. Tryptophan-Derived 3-Hydroxyanthranilic Acid Contributes to Angiotensin II-Induced Abdominal Aortic Aneurysm Formation in Mice In Vivo. Circulation. 2017; 136:2271–83. 10.1161/CIRCULATIONAHA.117.030972 28978552 PMC5716872

[5] Harden JL, Lewis SM, Lish SR, Suárez-Fariñas M, Gareau D, Lentini T, Johnson-Huang LM, Krueger JG, Lowes MA. The tryptophan metabolism enzyme L-kynureninase is a novel inflammatory factor in psoriasis and other inflammatory diseases. J Allergy Clin Immunol. 2016; 137:1830–40. 10.1016/j.jaci.2015.09.055 26725996 PMC4899291

[6] Williams DK, Markwalder JA, Balog AJ, Chen B, Chen L, Donnell J, Haque L, Hart AC, Mandal SK, Nation A, Shan W, Vite GD, Covello K, et al. Development of a series of novel o-phenylenediamine-based indoleamine 2,3-dioxygenase 1 (IDO1) inhibitors. Bioorg Med Chem Lett. 2018; 28:732–36. 10.1016/j.bmcl.2018.01.010 29398543

[7] Moyer BJ, Rojas IY, Murray IA, Lee S, Hazlett HF, Perdew GH, Tomlinson CR. Indoleamine 2,3-dioxygenase 1 (IDO1) inhibitors activate the aryl hydrocarbon receptor. Toxicol Appl Pharmacol. 2017; 323:74–80. 10.1016/j.taap.2017.03.012 28336214 PMC5495139

[8] Chinnadurai R, Copland IB, Patel SR, Galipeau J. IDO-independent suppression of T cell effector function by IFN-γ-licensed human mesenchymal stromal cells. J Immunol. 2014; 192:1491–501. 10.4049/jimmunol.1301828 24403533

[9] Sørensen RB, Hadrup SR, Svane IM, Hjortsø MC, Thor Straten P, Andersen MH. Indoleamine 2,3-dioxygenase specific, cytotoxic T cells as immune regulators. Blood. 2011; 117:2200–10. 10.1182/blood-2010-06-288498 21079151 PMC3062329

[10] Witkiewicz AK, Costantino CL, Metz R, Muller AJ, Prendergast GC, Yeo CJ, Brody JR. Genotyping and expression analysis of IDO2 in human pancreatic cancer: a novel, active target. J Am Coll Surg. 2009; 208:781–87. 10.1016/j.jamcollsurg.2008.12.018 19476837 PMC3176891

[11] Witkiewicz A, Williams TK, Cozzitorto J, Durkan B, Showalter SL, Yeo CJ, Brody JR. Expression of indoleamine 2,3-dioxygenase in metastatic pancreatic ductal adenocarcinoma recruits regulatory T cells to avoid immune detection. J Am Coll Surg. 2008; 206:849–54. 10.1016/j.jamcollsurg.2007.12.014 18471709

[12] von Bergwelt-Baildon MS, Popov A, Saric T, Chemnitz J, Classen S, Stoffel MS, Fiore F, Roth U, Beyer M, Debey S, Wickenhauser C, Hanisch FG, Schultze JL. CD25 and indoleamine 2,3-dioxygenase are up-regulated by prostaglandin E2 and expressed by tumor-associated dendritic cells in vivo: additional mechanisms of T-cell inhibition. Blood. 2006; 108:228–37. 10.1182/blood-2005-08-3507 16522817

[13] Terness P, Chuang JJ, Bauer T, Jiga L, Opelz G. Regulation of human auto- and alloreactive T cells by indoleamine 2,3-dioxygenase (IDO)-producing dendritic cells: too much ado about IDO? Blood. 2005; 105:2480–86. 10.1182/blood-2004-06-2103 15572592

[14] Prendergast GC, Malachowski WP, DuHadaway JB, Muller AJ. Discovery of IDO1 Inhibitors: From Bench to Bedside. Cancer Res. 2017; 77:6795–811. 10.1158/0008-5472.CAN-17-2285 29247038 PMC6021761

[15] Banerjee T, Duhadaway JB, Gaspari P, Sutanto-Ward E, Munn DH, Mellor AL, Malachowski WP, Prendergast GC, Muller AJ. A key in vivo antitumor mechanism of action of natural product-based brassinins is inhibition of indoleamine 2,3-dioxygenase. Oncogene. 2008; 27:2851–57. 10.1038/sj.onc.1210939 18026137

[16] Forouzandeh F, Jalili RB, Germain M, Duronio V, Ghahary A. Differential immunosuppressive effect of indoleamine 2,3-dioxygenase (IDO) on primary human CD4+ and CD8+ T cells. Mol Cell Biochem. 2008; 309:1–7. 10.1007/s11010-007-9635-y 18008147

[17] Okamoto T, Toné S, Kanouchi H, Miyawaki C, Ono S, Minatogawa Y. etc. Transcriptional regulation of indoleamine 2,3-dioxygenase (IDO) by tryptophan and its analogue. Cytotechnology. 2007; 54:107–13. 10.1007/s10616-007-9081-4 19003025 PMC2267499

[18] Soliman HH, Jackson E, Neuger T, Dees EC, Harvey RD, Han H, Ismail-Khan R, Minton S, Vahanian NN, Link C, Sullivan DM, Antonia S. A first in man phase I trial of the oral immunomodulator, indoximod, combined with docetaxel in patients with metastatic solid tumors. Oncotarget. 2014; 5:8136–46. 10.18632/oncotarget.2357 25327557 PMC4226672

[19] Orabona C, Grohmann U. Indoleamine 2,3-dioxygenase and regulatory function: tryptophan starvation and beyond. Methods Mol Biol. 2011; 677:269–80. 10.1007/978-1-60761-869-0_19 20941617

[20] Ogiso H, Ito H, Kanbe A, Ando T, Hara A, Shimizu M, Moriwaki H, Seishima M. The Inhibition of Indoleamine 2,3-Dioxygenase Accelerates Early Liver Regeneration in Mice After Partial Hepatectomy. Dig Dis Sci. 2017; 62:2386–96. 10.1007/s10620-017-4651-6 28639129

[21] Jochems C, Fantini M, Fernando RI, Kwilas AR, Donahue RN, Lepone LM, Grenga I, Kim YS, Brechbiel MW, Gulley JL, Madan RA, Heery CR, Hodge JW, et al. The IDO1 selective inhibitor epacadostat enhances dendritic cell immunogenicity and lytic ability of tumor antigen-specific T cells. Oncotarget. 2016; 7:37762–72. 10.18632/oncotarget.9326 27192116 PMC5122347

[22] Kristeleit R, Davidenko I, Shirinkin V, El-Khouly F, Bondarenko I, Goodheart MJ, Gorbunova V, Penning CA, Shi JG, Liu X, Newton RC, Zhao Y, Maleski J, et al. A randomised, open-label, phase 2 study of the IDO1 inhibitor epacadostat (INCB024360) versus tamoxifen as therapy for biochemically recurrent (CA-125 relapse)-only epithelial ovarian cancer, primary peritoneal carcinoma, or fallopian tube cancer. Gynecol Oncol. 2017; 146:484–90. 10.1016/j.ygyno.2017.07.005 28698009

[23] Yue EW, Sparks R, Polam P, Modi D, Douty B, Wayland B, Glass B, Takvorian A, Glenn J, Zhu W, Bower M, Liu X, Leffet L, et al. INCB24360 (Epacadostat), a Highly Potent and Selective Indoleamine-2,3-dioxygenase 1 (IDO1) Inhibitor for Immuno-oncology. ACS Med Chem Lett. 2017; 8:486–91. 10.1021/acsmedchemlett.6b00391 28523098 PMC5430407

[24] Mitchell TC, Hamid O, Smith DC, Bauer TM, Wasser JS, Olszanski AJ, Luke JJ, Balmanoukian AS, Schmidt EV, Zhao Y, Gong X, Maleski J, Leopold L, Gajewski TF. Epacadostat Plus Pembrolizumab in Patients With Advanced Solid Tumors: Phase I Results From a Multicenter, Open-Label Phase I/II Trial (ECHO-202/KEYNOTE-037). J Clin Oncol. 2018; 36:JCO2018789602. 10.1200/JCO.2018.78.9602 30265610 PMC6225502

[25] Beatty GL, O’Dwyer PJ, Clark J, Shi JG, Bowman KJ, Scherle PA, Newton RC, Schaub R, Maleski J, Leopold L, Gajewski TF. First-in-Human Phase I Study of the Oral Inhibitor of Indoleamine 2,3-Dioxygenase-1 Epacadostat (INCB024360) in Patients with Advanced Solid Malignancies. Clin Cancer Res. 2017; 23:3269–76. 10.1158/1078-0432.CCR-16-2272 28053021 PMC5496788

[26] Gibney GT, Hamid O, Lutzky J, Olszanski AJ, Mitchell TC, Gajewski TF, Chmielowski B, Hanks BA, Zhao Y, Newton RC, Maleski J, Leopold L, Weber JS. Phase 1/2 study of epacadostat in combination with ipilimumab in patients with unresectable or metastatic melanoma. J Immunother Cancer. 2019; 7:80. 10.1186/s40425-019-0562-8 30894212 PMC6425606

[27] Nayak-Kapoor A, Hao Z, Sadek R, Dobbins R, Marshall L, Vahanian NN, Jay Ramsey W, Kennedy E, Mautino MR, Link CJ, Lin RS, Royer-Joo S, Liang X, et al. Phase Ia study of the indoleamine 2,3-dioxygenase 1 (IDO1) inhibitor navoximod (GDC-0919) in patients with recurrent advanced solid tumors. J Immunother Cancer. 2018; 6:61. 10.1186/s40425-018-0351-9 29921320 PMC6009946

[28] Zhong S, Li W, Bai Y, Wu B, Wang X, Jiang S, Zhao Y, Ren J, Li H, Jin R. Computational study on new natural compound agonists of stimulator of interferon genes (STING). PLoS One. 2019; 14:e0216678. 10.1371/journal.pone.0216678 31120925 PMC6532845

[29] Zhou X, Yu S, Su J, Sun L. Computational Study on New Natural Compound Inhibitors of Pyruvate Dehydrogenase Kinases. Int J Mol Sci. 2016; 17:340. 10.3390/ijms17030340 26959013 PMC4813202

[30] Zhang F, Wen Q, Wang SF, Shahla Karim B, Yang YS, Liu JJ, Zhang WM, Zhu HL. Design, synthesis and antibacterial activities of 5-(pyrazin-2-yl)-4H-1,2,4-triazole-3-thiol derivatives containing Schiff base formation as FabH inhibitory. Bioorg Med Chem Lett. 2014; 24:90–5. 10.1016/j.bmcl.2013.11.079 24332628

[31] New Strategies to Advance Pre/Diabetes Care. Integrative Approach by PPPM. Mozaffari MS (editor). Springer Sciense and Business Media LLC; 2013 10.1007/978-94-007-5971-8

[32] Ohyama F, Tone S, Okamoto T, Shimoda K, Minatogawa Y. Introduction of indoieamine 2,3-dioxygenase in small intestine of mouse infected with parasitic helminth, Hymenolepis nana. Int Congr Ser. 2007; 1304:286–89. 10.1016/j.ics.2007.07.051

[33] Abstracts from 8th International ISSX Meeting. Drug Metab Rev. 2010; 39:1–388. 10.1080/03602530701532865

[34] Li CP, Ba HR, Jin K. Effect of Fe Doping on the Crystal Structures and Photoluminescence of ZnO Nanorods. Key Eng Mater. 2014; 636:105–09. 10.4028/www.scientific.net/KEM.636.105

[35] Le Floc’h N, Gondret F, Matte JJ, Quesnel H. Towards amino acid recommendations for specific physiological and patho-physiological states in pigs. Proc Nutr Soc. 2012; 71:425–32. 10.1017/S0029665112000560 22607969

